# Effects of BlueM^®^ against *Streptococcus mutans* biofilm and its virulence gene expression

**DOI:** 10.1590/0103-6440202305133

**Published:** 2023-03-06

**Authors:** Veronica Canela Estevam dos Santos, Patricia Milagros Maquera-Huacho, Maria Júlia Mancim Imbriani, Vivian M. Tellaroli Rodrigues Minhaco, Denise M. Palomari Spolidorio

**Affiliations:** 1 Department of Physiology and Pathology, School of Dentistry, São Paulo State University(Unesp), Araraquara, SP, Brazil; 2 Department of Diagnosis and Surgery, School of Dentistry, São Paulo State University (Unesp), Araraquara, São Paulo, Brazil.

**Keywords:** Streptococcus mutans, Biofilm, Virulence gene expression

## Abstract

This study evaluated the antimicrobial capacity of BlueM^®^ mouthwash against the bacterium *Streptococcus mutans* and its influence on *gbp*A gene expression as well as its cytotoxic effect on fibroblast cells. BlueM^®^ showed antimicrobial activity, with MIC and MBC values of 0.005% and 0.01%, respectively. The MBIC was 6.25% for *S. mutans*. CFU count and confocal microscopy revealed significant effect of BlueM^®^ on *S. mutans* biofilm pre-formed on dentin surfaces. Interestingly, the analysis of *gbpA* gene expression indicated a decrease in gene expression after 15 min of treatment with BlueM^®^ at a concentration of 25%. Moreover, BlueM^®^ exhibited low levels of cytotoxicity. In conclusion, our results showed the antimicrobial effectiveness of BlueM^®^ against *S. mutans*, its ability to modulate the expression of the *gbpA* gene and its low cytotoxicity. This study supports the therapeutic potential of BlueM^®^ as an alternative agent for the control of oral biofilm.

## Introduction

New substances are being developed in order to act on biofilm control, the prevention, and the progression of oral pathologies such as caries and periodontal disease. Among these products is BlueM^®^, which has in its formulation a compound based on oxygen, honey, and other compounds. Oxygen is important for the oral environment because it releases energy for chemical reactions and cellular functions, such as respiration, maintaining the redox balance, and preventing disturbances of redox homeostasis in the oral cavity ^1^. Regarding honey, its antimicrobial action suggests a possible production of hydrogen peroxide and other factors, including high sugar content, leading to some osmotic factors, and consequently causing bacterial dehydration and low pH ^2^.

Despite various approaches that have been used in an attempt to reduce the incidence of dental caries, this disease is still considered one of the most ubiquitous and costly biofilm-dependent oral diseases worldwide ^3^. Untreated caries lesions can have important consequences on oral health. Therefore, the trend is to move towards prevention and, there are several targets where prophylaxis can have effect ^4^. One of the main responsible for cariogenic biofilm formation of both enamel and dentinal is bacteria ^5^. Among them we can cite *Streptococcus mutans*, which is associated with the etiology of dental caries, causing the destruction of hard dental structures (enamel, dentin, and cementum) by the action of acidogenic/aciduric bacteria ^6^,^7^. It is widely accepted that the cariogenic potential of *Streptococcus mutans* is mainly associated with its ability to synthesize large amounts of extracellular glucan polymers from sucrose, which allows the permanent colonization of dental structures and the in situ formation of extracellular polymeric matrix ^8^.

Since bacterial adhesion is an important early event in bacterial colonization of tooth structures, different adhesion molecules have been characterized for bacterial species*.* In this process, extracellular enzymes of glucosyltransferase (Gtfs) glucan-binding proteins (gbps) and fructosyltransferase (ftf) are produced by *S. mutans*. Among them, *gbps* are virulence factors directly related to the ability of *S. mutans* to form dental biofilm. More specifically, *gbpA* is involved in the biofilm formation during the cariogenic process, adhesion to the structure, and the accumulation of microorganisms in the biofilm, resulting in its growth ^9^.

Clinically, the use of mouthwashes that contain fluoride, alcohols and antimicrobial agents (either chemical or natural) is recommended to assist in the control and reduction of dental biofilm. To be ideal, these antimicrobial agents should be effective against microorganisms, act quickly, maintain activity at low concentrations, have no side effects and be used without causing any discomfort ^10^. Despite having an excellent performance in chemical control, chlorhexidine - which is considered the gold standard for the reduction of oral pathogens - may promote changes in tooth color, desquamation of the oral mucosa, sensitivity and taste alteration ^11^,^12^.

Epidemiological surveys show that dentin exposure to the oral cavity is becoming common as the population ages ^13^. In this sense, there is increasing interest in evaluating the antibiofilm and antibacterial effect of new products on biofilm formation in dentin. Thus, BlueM^®^ mouthwash presents itself as an alternative to conventional methods for biofilm control. However, its properties and mechanisms of action in cariogenic biofilm are not clarified in the literature yet. Thus, the aim of this study is to investigate the antimicrobial potential of this product against *S. mutans* biofilm dentin formation and its ability to influence *gbpA* expression. Moreover, the biocompatibility of this mouthwash with fibroblasts is also evaluated.

## Material and methods

### Solutions

Blue M^®^ (BlueM^®^, Curitiba, PR, Brazil) was serially diluted (50% - 0.002% v/v) and prepared in BHI medium (Brain Heart Infusion, Acumedia, Lansing, MI, USA). Chlorhexidine stock solution (CHX 2%) was used as a positive control and prepared at 0.12%.

### Bacterial conditions

*Streptococcus mutans* (ATCC 25175) was used as a standard strain. Microbial suspension of *S. mutans* was prepared from culture previously grown in BHI (Brain Heart Infusion, Acumedia, Lansing, MI, USA) and incubated at 37 °C for 18 h in a 5% CO_2_ atmosphere.

### Determination of Minimum Inhibitory Concentration and Minimum Bactericidal Concentration

The broth microdilution method was used to determine the Minimum Inhibitory Concentration (MIC) and Minimum Bactericidal Concentration (MBC). Briefly, serial dilutions of Blue M^®^ were prepared and distributed onto 96-well plates, followed by the addition of a bacterial suspension of *S. mutans*. The final inoculum concentration of *S. mutans* was 1.0 x 10^5^ CFU mL^-1^ per well. The microplates were incubated at 37 ºC in a 5% CO_2_ atmosphere for 24 h. BHI medium served as a negative control, while chlorhexidine 0.12% was used as a positive control. The bacterial growth was monitored by absorbance at 600 nm using a micro plate reader (Synergy H1 Multi-Mode Reader - BioTek, Winooski, VT, USA). The MIC was defined as the lowest Blue M^®^ concentration without bacterial growth. The MBC was obtained after placing 10 μL aliquots from each well on Mitis Salivarius Agar (Agar Mitis Salivarius, Himedia, Vadhani, MB, IND) culture medium and incubating them at 37 ºC for 48 h. The MBC was defined as the lowest concentration capable of inhibiting bacterial growth.

### Minimum biofilm inhibitory concentration (MBIC)

*S. mutans* biofilms were formed in 96-well plates, as described by Maquera-Huacho et al. ^(14)^, with some modifications. Unstimulated human saliva (CAAE 41872620.0.00005416) was collected from a healthy volunteer (male, 26 years old), who claimed not to have used antibiotics or any other medications at least three months before the collection. The saliva was centrifuged and the supernatant was filtered using a 0.22 μm membrane filter (Corning Inc., Corning, NY, USA) ^(15)^. First, 50 μL of the sterile human saliva was added to each well and incubated at 37 °C under gentle shaking (70 rpm) for 2 h. After the incubation period, the saliva was removed and 200 μL of *S. mutans* (1×10^5^ CFU mL^-1^) was inoculated for biofilm formation. Then, the plates were incubated for 24 h at 37 ^o^C in a 5% CO_2_ atmosphere. Afterward, planktonic bacteria were removed and the biofilms were exposed to 200 μL of different concentrations (50% - 0.01% v/v) of BlueM^®^. After 24 h, the biofilm biomass was assessed by crystal violet (CV) dye, as described by Maquera Huacho et al ^(16)^. Briefly, the biofilms were fixed with 100 μl of 96% EtOH for 20 min, followed by drying at room temperature. The biofilms were subsequently stained with 100 μL of the CV solution (1%) for 15 min, washed with PBS to remove the unbound CV dye and dried at room temperature. The CV was solubilized through the addition of 75 μL of acetic acid (5%) for 45 min prior to measuring the absorbance at 630 nm (OD_630_, Synergy H1 Multi-Mode Reader - BioTek, Winooski, VT, USA).

### Biofilm formation on specimens

*S. mutans* biofilms were grown on sterile dentine surface. Previously, bovine teeth were prepared as described by Bordini et al. ^9^. The dentin specimens (8.0 mm x 3.0 mm x 1.5 mm) were polished, submitted to surface roughness (Ra) evaluation by a portable rugosimeter (Mitutoyo surftest SJ-401, Mitutoyo Corporation, Japan), cleaned and sterilized with ethylene oxide. Subsequently, the specimens were placed in 48-well plates, and the salivary pellicle was preformed using sterile human saliva, as previously described. Dentin discs were infected with bacterial suspension of *S. mutans* (1.0 x 10^5^ CFU mL^-1^) in BHI broth and incubated at 37 °C for 24 h for biofilm formation. Afterwards, the medium was gently aspirated and the biofilms were treated for 60 s with 500 µL per well of BlueM^®^ concentrations (50%, 25% and 12.5%). Then, all discs were gently washed with PBS and transferred to sterile Eppendorf tubes with 1 mL of physiological saline. The discs were harvested by vigorous vortexing (1 min). The resulting adherent bacteria were diluted and plated on Mitis Salivarius Agar for 48 h at 37 °C, while the resulting biofilm was evaluated by Colony Forming Units per milliliter (CFU mL^-1^).

### Confocal laser scanning microscopy (CLSM)

To evaluate CLSM images, biofilms grown on dentin discs and treated with different concentrations of BlueM^®^ were washed and labeled with Live/Dead BacLight Bacterial Viability Kit (L7012, Invitrogen Molecular Probes, Eugene, OR, USA) containing SYTO9 (SY) and propidium iodide (PI), according to the manufacturer’s instructions. The excitation/emission wavelengths were 488/500 nm for SYTO and 488/635 nm for PI. Fluorescence from the stained cells was observed using a confocal laser scanning microscope (LSM 780 inverted, Zeiss, Jena, Germany), and the images were acquired with ZEN 2012 software (Zeiss) at a resolution of 1,024 by 1,024 pixels. Single focal plane images of the biofilm were captured by the system with a 20x magnification lens. The area selected for the biofilm analysis was randomly defined, but not so close to the edges of the specimens.

### RNA isolation and quantitative RT-PCR

To investigate the influence of BlueM^®^ treatment on *gbpA* (glucan-binding protein A) gene expression, *S. mutans* was grown in BHI medium until its mid-log phase (OD_600_ *S. mutans* = 0*.*45), followed by the quick addition of different concentrations of BlueM^®^ (50% and 25%) and incubation for 15 and 30 min at 37 °C. Control cells were incubated in the absence of BlueM^®^, and CHX 0.12% was used as a positive control. Bacteria were collected by centrifugation (7,000g, 5 min) and treated for 5 min with RNAprotect Bacteria (Qiagen GmbH, Hilden, Germany). The bacteria were lysed and the RNA was isolated using RNeasy minikit (Qiagen GmbH, Hilden, Germany). The amounts of mRNA were quantified using Synergy H1 (Biotek, Winooski, VT, USA). The reverse transcription polymerase was performed using a High Capacity cDNA Reverse Transcriptions Kit (Applied Biosystems, Foster City, Calif., USA) and 100 ng mL^-1^ of the RNA sample. Then, the obtained cDNA was used in the Real-time PCR reactions to determine the quantity of *gbpA* target gene and the constitutive 16S rRNA gene, used as internal control for data normalization. The primers used for RT-PCR were purchased from Life Technologies Inc. (São Paulo, SP, Brazil) and are listed in Box 1. Triplicate reactions were prepared using Fast SYBR™ Green Master Mix. The samples were amplified using StepOnePlus Real-Time PCR System equipment (Applied Biosystems, USA) followed by the detection of amplification conditions (95 °C for 20 s, 40 cycles at 95 °C for 3 s and 60 °C for 30 s), and analyzed using StepOne 2.1 software (Applied Biosystems, USA).

**Box 1:**
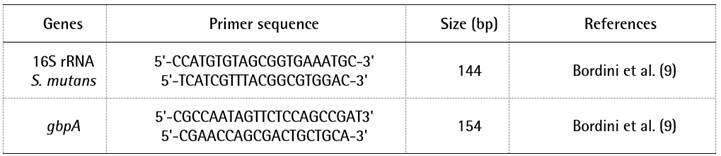
Primers for RT-PCR analysis of *S. mutans*

### Biocompatibility

Fibroblast cell lines (L929) were cultured in Dulbecco's Modified Eagle Medium (DMEM) (Sigma Chemical Co., St. Louis, MO, USA) supplemented with 10% fetal bovine serum (Gibco, Grand Island, NY, USA) and 1% penicillin, streptomycin and glutamine (100 UT/mL penicillin, 100 μg/mL streptomycin and 2 mmol/L glutamine (Gibco, Grand Island, NY, USA) at 37 °C in a humidified atmosphere containing 5% CO_2_. Fibroblasts were seeded at a density of 2 x 10^4^ cells/well into 96-well plates (100 μL), and cultivated until confluence. Afterwards, the cells were treated with different concentrations of BlueM^®^ (50% - 0.04% v/v) and prepared in supplemented DMEM medium for 2 h. Camptothecin 10 µM was used as a positive control of the experiment, whereas DMEM medium was used as a negative control. To determine cell viability, MTT assay (3-(4,5- dimethylthiazol-2-yl)-2,5-diphenyltetrazolium bromide - Sigma-Aldrich) was performed ^18^. Briefly, the culture medium was aspired and the MTT solution (5 mg mL^-1^) was added and incubated for 4 h. Then, the acidified isopropanol solution (100 µL, 0.04 N HCl) was used to dissolve the purple formazan crystals. Spectrophotometry (570 nm) using ELISA plate reader (OD630, Synergy H1 Multi-Mode Reader - BioTek, Winooski, VT, USA) was used to evaluate the cell viability.

### Statistical analysis

All assays were performed in triplicate in three independent experiments, and the data normality was tested by Shapiro-Wilk test before analysis. The significance level was set at 95% (*p* <0.05). One-way ANOVA with Tukey's *post-hoc* test for paired comparisons was used to compare the results of each outcome according to the different concentrations of BlueM^®^. The data obtained were analyzed using GraphPad Prism 9.0 (GraphPad Software Inc., USA).

## Results

### Antibacterial activity and biofilm inhibition

Table 1 shows the MIC, MBC and MBIC values for the concentrations of BlueM^®^ tested in planktonic cultures and biofilm of *S. mutans.* After the analysis of serial dilutions of BlueM^®^ (50% - 0.002% v/v), it was possible to observe that MIC and MBC values were 0.005% and 0.01%, respectively. The effectiveness of biofilm inhibition using different concentrations of BlueM^®^ was analyzed by crystal violet test. Finally, the MBIC value of BlueM^®^ was 6.25%.

**Table 1:** Minimum inhibitory concentration (CIM), Minimum Bactericidal Concentration (MBC), and Minimum biofilm inhibition concentration (MBIC) of BlueM^®^ on *S. mutans*

	BlueM^®^		
Microorganism	MIC (%)	MBC (%)	MBIC (%)
*S. mutans*	0.005	0.01	6.25

### Inhibition of biofilm formation

**Figure 1:**
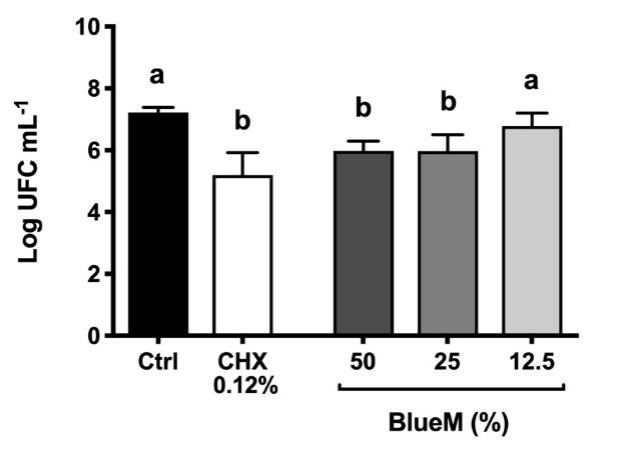
Effect of BlueM^®^ on *S. mutans* (ATCC 25175) biofilm grown on bovine dentin surfaces. Bacteria counts after each treatment are expressed as logarithm of colony forming units (CFU) per milliliter. Statistical differences are represented by different letters for comparison between treatments (One-way ANOVA followed by Tukey's test, *p<0*.05).

The effect of BlueM^®^ against *S. mutans* biofilm pre-formed on dentin specimens was evaluated after 60 s of treatment (Figure 1). Concentrations of 50% and 25% of BlueM^®^ showed a statistically significant decrease in the number of viable microorganisms when compared to the negative control group. On the other hand, the concentration of 12.5% of BlueM^®^ did not promote a statistically significant decrease (*p>0*.05%).

### Confocal laser scanning microscopy (CLSM)

**Figure 2:**
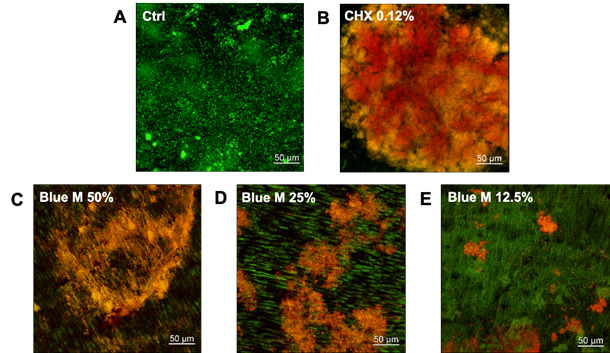
Confocal laser scanning microscopy images of BlueM^®^ action on *S. mutans* ATCC 25175 biofilm developed on dentin specimens. Negative Control (A), Chlorhexidine 0.12% (B), BlueM^®^ 50% (C), BlueM^®^ 25% (D), and BlueM^®^ 12.5% (E).

Figure 2 shows CLSM images of *S. mutans* biofilm pre-formed on dentin and treated with BlueM^®^, where it can be seen that the stained live and dead cells emit green and red fluorescence signals, respectively. The untreated control (Figure 2A) shows a homogeneous mass of viable microorganisms with green fluorescence emission. The biofilm treated with CHX 0.12% displays an extensive red fluorescence and a green fluorescence in the background, indicating less viability of cells (Figure 2B). A predominance of red/orange coloration can be observed, indicating a reduction on the viability of microorganisms after treatment with BlueM^®^ 50% and 25% (Figures 2C and 2D, respectively). More specifically, Figure 2C shows a great red fluorescence area, confirming that BlueM^®^ 50% was able to eradicate the *S. mutans* biofilm. On the other hand, BlueM^®^ 25% showed a less pronounced effect on biofilm than BlueM^®^ 50% (Figure 2D). Regarding the biofilms treated with the BlueM^®^ 12.5% (Figure 2E), it can be seen that they emit green fluorescence, implying a greater number of viable bacteria.

### Gene expression assays

**Figure 3:**
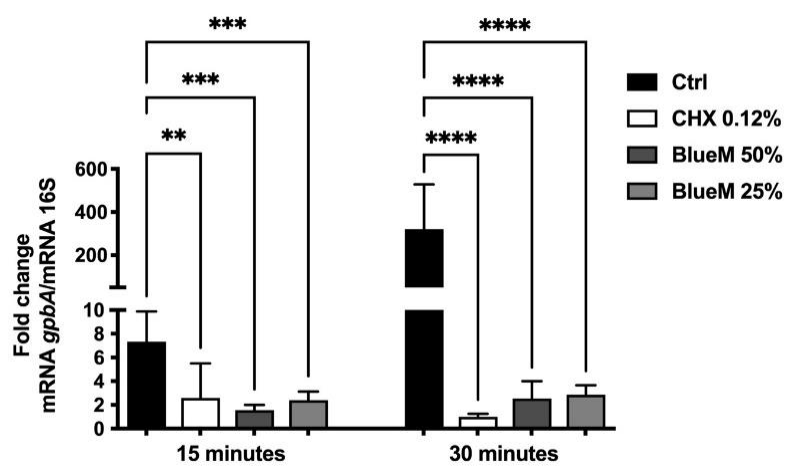
Effect of BlueM^®^, on mRNA expression of the *gbpA* gene involved in adhesion and biofilm formation of *S. mutans* (ATCC 25175). Results are expressed as means ± SD of triplicate assays for three independent experiments (ANOVA/Tukey's test, α = 0.05). ^┌─┐^indicates statistically significant difference for each evaluation period (**p<0.01, ***p<0.001, ****p<0.0001).

The expression of the *gbpA* gene, related to bacterial adhesion and dental biofilm formation by *S*. *mutans* was evaluated after 15 and 30 min of treatment with BlueM^®^ 50% and 25%. As shown in Figure 3, a highly significant reduction in the *gbpA* gene expression (***p<0.001, ****p<0.0001) was observed after treatment with BlueM^®^ in both periods evaluated. Similarly, chlorhexidine 0.12% showed a significant reduction in gene expression (**p<0.01, ****p<0.0001). However, chlorhexidine did not show statistical difference compared to BlueM^®^ 50% and 25% after 15 and 30 min (*p*>0.05).

### Biocompatibility

**Figure 4:**
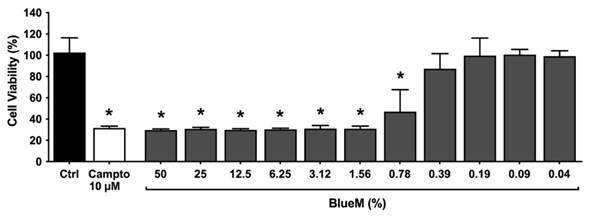
Cell viability (% of control) of L929 fibroblasts after 2h of exposure with different concentrations of BlueM^®^ (50% - 0.04% v/v) and 10 μM camptothecin used as positive control. *, indicates statistically significant reduction in cell viability compared to the control group (One-way ANOVA followed by Tukey's test, *p* <0.001).

The viability of L929 fibroblast cells after treatment with different concentrations of BlueM^®^ is shown in Figure 4. The number of cells in the control group was set at 100%. It can be observed that concentrations from 50% to 0.78% of BlueM^®^ showed statistically significant decrease (*p<*0.01%) in cell viability after 2 h of treatment when compared to the control group. However, the same behavior was not observed with concentrations from 0.39% to 0.04% (*p>0*.01%).

## Discussion

The use of complementary methods for oral hygiene associated with traditional methods (i.e., mouthwashes) has been widely studied in order to understand the possible mechanisms of control of pathogenic bacteria in the oral cavity. In the present study, we evaluated for the first time the antimicrobial effect of BlueM^®^ against *S. mutans* and its biocompatibility using fibroblasts.

Dental caries is a multifactorial disease, which depends on the presence of biofilm accumulation and diet; it presents itself as a dynamic disease that results in the loss of minerals from dental tissues ^17^. The main bacterium associated with this disease is *S. mutans*, which in the presence of fermentable carbohydrates releases acid on the tooth surface as a metabolic product ^18^. The cariogenic potential of *S. mutans* is related to its ability to synthesize extracellular polymers, such as glucan, to convert carbohydrates into organic acids and to maintain itself at low pH ^8^.

Currently, new substances are being developed with the aim of controlling biofilm formation and preventing the progression of oral pathologies such as caries. BlueM^®^ presents itself as a commercial product with antimicrobial effect that uses active oxygen (its main constituent) as an alternative means for microbiota control. Taking this into account, Cunha et al. ^19^ ^)^ and Shibli et al. ^20^ evaluated the effectiveness of BlueM^®^ on inhibiting supragingival biofilm formation and demonstrated the antibiofilm effect of active oxygen. Thus, it was found that BlueM^®^ has antioxidant and antimicrobial properties, in addition to being an important source for the discovery of new effective compounds for caries. Nonetheless, there are no studies in the literature regarding the effect of BlueM^®^ against cariogenic bacteria or cariogenic biofilm.

In order to fill this gap, this study aimed to present the antimicrobial effect of BlueM^®^ against *S. mutans* biofilm. As showed in Table 1, the MIC and MBC values were 0.005% and 0.01%, respectively, demonstrating that low concentrations of BlueM^®^ were able to both inhibit bacterial growth and show bactericidal action. No studies were found in the literature about the antimicrobial potential of BlueM^®^ against cariogenic bacteria such as *S. mutans* in planktonic or biofilm form. MBIC evaluation was used as a preliminary test to determine the lowest concentration capable of inhibiting 50% of *S. mutans* biofilm formation. Although the crystal violet test is considered a quantitative test to evaluate biofilm biomass, it has been commonly used as an evaluation method to determine biofilm reduction and/or inhibition ^16^. Zhenbo et al. ^21^ ^)^ quantified microbial biofilm formation by correlation analysis between crystal violet (CV) and XTT assays. The results showed that both trials were effective and beneficial. Our results suggest that BlueM^®^ 6.25% was able to reduce the biofilm of *S. mutans*, which is in agreement with the results reported by Cunha et al. ^19^.

During biofilm formation, tooth surfaces are immediately covered by salivary mucoproteins, forming the acquired film; this is an important factor for bacterial adhesion through ionic bonds - which are initially weak and later become stronger through the interaction between bacterial molecules ^22^. Therefore, since saliva is considered an important and essential factor for biofilm formation, human saliva was used in the present study in order to simulate a clinical condition. There are several models for the development of biofilm in vitro, however, simplified biofilm models that use a single bacterial species appear to be useful to study the physiological activities of specific bacteria, as well as obtain a quick analysis of the results, and with low cost of implementation, when compared to more complex models ^23^. The dentin biofilm model used in the present investigation was based in Bordini et al. ^9^. Additionally, in vitro models using tooth substrates are able to analyze and understand the interactions of bacterial metabolites with enamel or dentine structure ^24^.

The primary colonizers in the biofilm are predominantly Gram-positive bacteria of the genus *Streptococcus*, which are also responsible for the secondary bacterial colonization, making the biofilm more complex and mature ^25^,^26^. For having a diameter of 0.7-0.9 μm, *S. mutans* has its penetration inside dentinal tubules (1.5-2.0 μm in diameter) facilitated ^9^ ^,^ ^27^. As observed in the results of CFU mL^-1^ and CLSM images (Figures 1 and 2), there was a statistically significant reduction in *S. mutans* biofilm after treatment with Blue M^®^ in bovine dentin specimens*.* In this context, it can be suggested that the antimicrobial effect of BlueM^®^ is due to its compound, active oxygen or other compounds as sodium perborate and honey. According BlueM^®^ International, the mechanism of action of its products is based on the controlled delivery of several reactive oxygen species (ROS). Hollar et al. ^28^ described that the oral care product BlueM^®^ contains sodium perborate and honey. Then, in the presence of water, sodium perborate decomposes into hydrogen peroxide (H_2_O_2_) and sodium borate, both with antiseptic effects. On the other hand, honey sugars are also converted to hydrogen peroxide and gluconolactone under the influence of the enzyme glucose oxidase ^29^,^30^. As is known, hydrogen peroxide has the ability to spontaneously dissociate into several reactive oxygen species for lipid peroxidation of bacterial cell walls and in this way, it is considered a relevant broad range and nonspecific antibacterial agent ^19^.

One of the mechanisms by which the virulence factor influences on the colonization process of *S. mutans,* is the fact that its expression of some genes responsible for bacterial accumulation and growth on the tooth surface ^31^. As previously described, *Gbps* are binding proteins that participate in biofilm formation, allowing adhesion of other bacterial cells due to their action as cell surface receptors for glucans synthesized from sucrose ^32^,^33^. Particularly, *S. mutans* synthesizes three glucan-binding proteins with no known enzymatic activities: *gbpA, gbpB and gbpC* ^32^,^33^. The virulence-associated properties of these specific genes are still under study. More specifically, *gbpA* is the main representative of this group, being a glucan-dependent gene that acts on bacterial adhesion and cohesion during biofilm formation ^31^.

Biofilm formation is influenced by bacterial communication through the quorum-sensing signaling system ^34^. Extracellular glucans promote adhesion and are critical for increasing the concentration of *S. mutans* in the biofilm ^35^. In the present study, qPCR was then performed to investigate the effect of BlueM^®^ on the adhesion-related gene expression of *S. mutans*. Our results showed that the treatment of *S. mutans* biofilm with BlueM^®^ reduced the expression of *gbpA*. These results support the positive effect of BlueM^®^ on *S. mutans* biofilm presented in Figures 1 and 2, leading us to suggest that changes in virulence may consequently modify the biofilm structure. In this context, Jang et al. ^36^ ^)^ suggest that the expression of the *gbpA* gene was possibly correlated with the number of microorganisms present in the biofilm, that is, it decreased as a function of the number of microorganisms.

Finally, the biocompatibility of a product intended for the treatment or prevention of oral infections is an important parameter to be considered in terms of cell survival. Thus, cytotoxicity tests of BlueM^®^ were performed on fibroblast-like cells. These types of cells were selected because they are found in the composition of oral mucosa, which is the first barrier that a mouthwash comes into contact with. In this study, lower concentrations of BlueM^®^ did not show cytotoxic effects on fibroblasts cells. Mattei et al. ^37^ who used low concentrations of BlueM® (1 μl/mL equivalent to 1%) on keratinocytes cells and suggested that higher concentrations of BlueM® may present cytotoxic effects reported similar results. This can be explained by the fact that at high concentrations, active oxygen promotes cell death, but at low concentrations, it is able to regulate microbial colonization, immune response and cell function ^38^. No studies evaluating the effect of this product on fibroblasts were found.

## CONCLUSION

The present study showed that BlueM^®^ has antibiofilm activity against *S. mutans*, presenting itself as a biocompatible product. This product has an antimicrobial effect against *S. mutans*, promoting bactericidal, bacteriostatic, antibiofilm, and non-cytotoxic effects at low concentrations. Moreover, BlueM^®^ is able to reduce the expression of the *gbpA* gene, interfering with the adhesion capacity of *S. mutans*. However, additional in vivo and in vitro tests are still needed to corroborate and elucidate its mechanism of action.
